# Temporal dynamics of sequential motor activation in a dual-prime paradigm: Insights from conditional accuracy and hazard functions

**DOI:** 10.3758/s13414-020-02010-5

**Published:** 2020-03-12

**Authors:** Maximilian P. Wolkersdorfer, Sven Panis, Thomas Schmidt

**Affiliations:** Faculty of Social Sciences, Experimental Psychology Unit, University of Kaiserslautern, Erwin-Schrödinger-Str. Geb. 57, D-67663 Kaiserslautern, Germany

**Keywords:** Feedforward sweep, Response priming, Event history analysis, Reaction- time analysis, Visuomotor

## Abstract

In response priming experiments, a participant has to respond as quickly and as accurately as possible to a target stimulus preceded by a prime. The prime and the target can either be mapped to the same response (consistent trial) or to different responses (inconsistent trial). Here, we investigate the effects of two sequential primes (each one either consistent or inconsistent) followed by one target in a response priming experiment. We employ discrete-time hazard functions of response occurrence and conditional accuracy functions to explore the temporal dynamics of sequential motor activation. In two experiments (small-*N* design, 12 participants, 100 trials per cell and subject), we find that (1) the earliest responses are controlled exclusively by the first prime if primes are presented in quick succession, (2) intermediate responses reflect competition between primes, with the second prime increasingly dominating the response as its time of onset is moved forward, and (3) only the slowest responses are clearly controlled by the target. The current study provides evidence that sequential primes meet strict criteria for sequential response activation. Moreover, it suggests that primes can influence responses out of a memory buffer when they are presented so early that participants are forced to delay their responses.

Priming paradigms are very popular in many fields of cognitive psychology to study how exposure to a prime stimulus influences the response to a subsequently presented target stimulus. In general, the representations that mediate priming can be located at perceptual (Wiggs & Martin, 1998), conceptual/semantic (e.g., Schacter & Buckner, 1998), lexical (e.g., Fernández-López, Marcet, & Perea, 2019), phonological (e.g., Ferrand & Grainger, 1992), and/or motor response levels (e.g., Rosenbaum, 1983). In this paper we focus on the so-called response priming paradigm (Klotz & Neumann, 1999; Klotz & Wolff, 1995; Vorberg, Mattler, Heinecke, Schmidt, & Schwarzbach, 2003). In a typical response priming experiment, a participant has to respond as quickly and as accurately as possible to a target stimulus preceded by a (masked or unmasked) prime stimulus. The prime and the target can either be mapped to the same response (consistent trial) or to different responses (inconsistent trial). While consistent trials typically show accelerated and more accurate responses, inconsistent trials show decelerated and less accurate responses, respectively. The differences between consistent and inconsistent trials in both mean reaction times (RTs) and overall error rates (ERs) define the *response priming effect*. Characteristically, this priming effect increases linearly with stimulus-onset asynchrony (SOA) for SOAs of up to about 100 ms (Vorberg et al., 2003). Response priming effects are believed to be mostly mediated by motor response conflicts (Schmidt, Haberkamp, & Schmidt, 2011; Schmidt, 2002). However, how a rapid sequence of visual stimuli is processed and converted into motor action is still under debate. In order to gain insights into the covert temporal dynamics of our visual system and the online transfer of visual signals into overt behavior, we employ event history analysis, a longitudinal technique to perform a distributional analysis.

## Multiple-prime paradigm

What if instead of only one prime, a sequence of primes is preceding a target stimulus? A number of previous studies have touched upon this question. Jaśkowski, Skalska, and Verleger (2003) presented five pairs of squares sequentially, with an SOA of 35 ms, so that each stimulus masked the previous one via metacontrast. The last and largest pair was the target, and observers had to decide whether the left or right square contained a gap. The first four pairs could serve as masked primes that contained a gap in the same (consistent) or opposite (inconsistent) square as the target. They found that the priming effect in mean correct RT increases with the number of primes presented in a sequence of successively masked stimuli. Because all of the primes within a single trial were either consistent or inconsistent to the target, this result would be expected from the accumulation of prime information (Miller, 1982). Jaśkowski et al. (2003) concluded that “motor activation evoked by a series of primes does accumulate, facilitating or inhibiting motor responses to the target” (p. 913).

Similarly, Breitmeyer and Hanif (2008) showed that when two successively presented prime stimuli are both consistent to a target in terms of shape (square versus diamond), mean RTs are faster than when only one of the two primes is consistent. Furthermore, they found that the priming effects from the second prime dominate over those of the first prime. That is, if the first prime was consistent and the second inconsistent to the target (condition “CI”), mean RT increased much more than when the first prime was inconsistent and the second consistent (condition “IC”). This contradicts the idea that due to the longer Prime1-target SOA, the first prime should cause a larger priming effect than the succeeding second prime. They argue that the second prime instead updates and overrides the effects of the first prime.

Grainger, Scharnowski, Schmidt, and Herzog (2013) employed two 20-ms Vernier stimuli as primes. In a series of experiments, they found that (1) two primes presented in immediate succession at the same location integrate before activating a motor response, and do not cause sequential activation; (2) two identical primes yield larger priming effects than single primes; (3) one consistent and one inconsistent prime presented simultaneously at different locations cancel each other’s effects. More importantly, in the varying-primes condition of their Experiment 3, they presented two lateralized Vernier primes and a central Vernier target, kept the Prime 1–target SOA constant at 200 ms, and varied the interprime interval (and thus also Prime 2–target ISI). For interprime intervals of 30 and 80 ms Prime 2 clearly dominated, but for an interprime interval of 150 ms (and a corresponding Prime 2–target ISI of 30 ms) Prime 1 dominated slightly. The authors propose that all visual stimuli enter a time-selective buffer stage, integrate, and only then initiate a motor response. Instead of activating their associated responses in strict sequence, their joint impact is determined by their relative dominance in the motor buffer.

However, it has been suggested that—in the context of response-conflict paradigms such as response priming and flanker effects—sequential visual stimuli elicit sequential feedforward sweeps (Bullier, 2001; Lamme & Roelfsema, 2000; VanRullen & Koch, 2003). These fast and bottom-up processes can activate motor responses in a strictly sequential manner (T. Schmidt et al., 2011). Moreover, since both prime and target in a response priming paradigm activate their respective motor responses, response conflict arises if prime and target are inconsistent, thus leading to an increase in RT (Schmidt, 2014). Several studies have demonstrated the existence of this feedforward and sequential activation, in both neuronal activity, such as lateralized readiness potentials (Eimer & Schlaghecken, 1998; Vath & Schmidt, 2007), and overt behavior, such as the time course of pointing movements (Schmidt & Schmidt, 2010; Schmidt, 2002; Schmidt & Schmidt, 2009) and response-time distributions (Panis & Schmidt, 2016). In particular, these studies demonstrated that the first responses are exclusively triggered by prime properties, independent of the target, whereas only later responses are influenced by target properties.

Schmidt, Niehaus, and Nagel (2006) hence proposed a *chase theory of response priming* in which they formulated the *chase criteria* of such a feedforward system: (1) Prime rather than target signals determine the onset and initial direction of the response; (2) target signals influence the response before it is completed; (3) movement kinematics initially depend on prime characteristics only and are independent of all target characteristics (see Schmidt, 2014, for precise definitions of criteria and predictions). Such a simple feedforward-sweep model seems to account very well for response priming effects at short SOAs (up to 100 ms), but would predict unrealistically high error rates for longer SOAs (because in inconsistent trials, the prime would always have enough time to drive the wrong response to completion). Therefore, priming effects at longer SOAs are more plausibly carried by the content of a response buffer that carries information from both primes, but is dominated by the second one (Grainger et al., 2013). This buffer would allow participants to delay their responses, waiting out the target.

## Event history analysis

The aims of the current study were to trace sequential priming effects over the time course of a trial to see (a) whether sequential primes actually initiate sequential response activation, (b) whether that sequence conforms to the chase criteria at short SOAs, and (c) how the influence of the first prime changes when the interprime interval is prolonged. In order to investigate the temporal dynamics of response activation, one must take the passage of time into account when analyzing behavioral output. Here, we make use of a relatively new approach to analyze reaction time data: Event history analysis (EHA: Allison, 1982, 2010; Luce, 1986; Panis & Schmidt, 2016; Singer & Willett, 2003). In EHA, it is assumed that for each time point since target onset in each trial of an experiment, there is a risk for the response to occur. The time after target onset is subdivided into a series of nonoverlapping and contiguous time bins indexed by t, t ∈ {1…*n*}, and for each time bin, the discrete-time *hazard* probability of response occurrence is estimated. The hazard probability *h*(t) is defined as the conditional probability that a response occurs sometime within bin t given that no response has been emitted in previous bins: *h*(t) = P(T = t | T ≥ t) (Allison, 1982, 2010; Luce, 1986; Panis, Torfs, Gillebert, Wagemans, & Humphreys, 2017; Panis & Wagemans, 2009). The survival function *S*(t) = *P*(T > t) estimates the probability that no response has been emitted by the time Bin t is completed. In addition, *P*(t) = *P*(T = t) gives the unconditional probability that a response (no matter whether correct or incorrect) occurs within Bin t.^1^ Since correct and incorrect response occurrences are not independent (Burle, Vidal, Tandonnet, & Hasbroucq, 2004; Praamstra & Seiss, 2005), we calculate the conditional accuracy *ca*(t) = *P*(correct response | T = t), the probability that a response emitted in time Bin t is correct. Together, *h*(t) and *ca*(t) give an unbiased description of the time course of the latency and accuracy of responses (Panis & Hermens, 2014; Panis & Schmidt, 2016).

## Current study

Here, we investigate the effects of two sequential primes followed by one target on response occurrence and accuracy in a response priming experiment. Our goal was to investigate (a) whether sequential primes actually initiate sequential response activation, or integrate in a buffer before a response is emitted, (b) whether that response activation sequence conforms to the rapid-chase criteria at short SOAs, and (c) how the influence of the first prime changes when the SOAs are all prolonged.

We designed a stimulus layout where two primes can be presented in sequence without mutual interference and without masking. Further, we varied the timing of the stimuli by keeping the Prime 1 target (P1-T or SOA1) SOA fixed and moving the onset of Prime 2, resulting in different combinations of Prime 1–Prime 2 (P1–P2) and Prime 2–target (P2–T or SOA2) SOAs. Each prime could either be consistent or inconsistent to the target. In a first experiment we investigated quick successions of primes and target, a second experiment used prolonged stimulus-onset asynchronies. We reasoned from the idea that when the P1–T SOA is short (Experiment 1), participants can rely on feedforward response activation and give speeded responses without using the response buffer. In contrast, when the P1–T SOA is long (Experiment 2), participants are forced to withhold responses in order to avoid errors triggered by inconsistent primes, and in that situation the response buffer can influence the response.

## Experiment 1

### Method

**Fig. 1:**
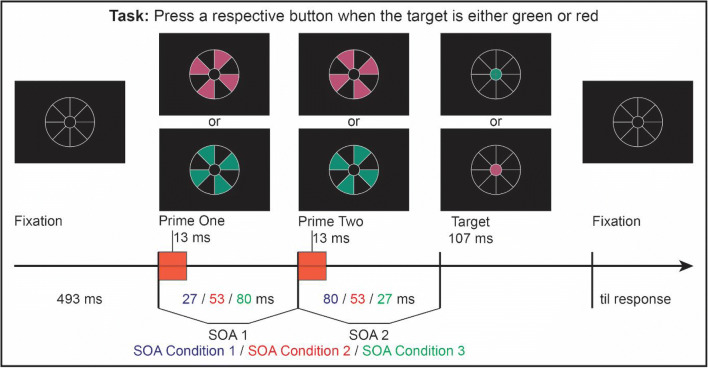
Stimulus displays and design. After fixating the center of the white lollipop frame, a sequence of two primes and a target is presented, with SOA1–SOA2 combinations of 27/80, 53/53, or 80/27

We constructed a stimulus arrangement dubbed the ‘lollipop’ that allows us to present a sequence of primes and targets without any spatial overlap or masking (see Fig. 1). The lollipop consisted of a large circle subdivided into eight segments that would contain the primes. A circle in the center of the lollipop contained the target and served as fixation point. Participants were instructed to give speeded responses to the color of the target—red or green—with two successive primes appearing prior to its onset. For the first prime, every other lollipop segment briefly changed color simultaneously (all either red or green). For the second prime, the previously unoccupied segments all briefly turned red or green simultaneously, independent of the color of the first prime.

#### Participants

Twelve participants (seven female, ages 22–36 years, *M* = 28.2 years) were recruited out of the pool of students of the University of Kaiserslautern. They participated in one 60-minute session for each experiment and were rewarded with course credits. All of them had normal or corrected-to-normal vision (17% with correction). Each participant gave informed consent and was treated in accordance with the ethical standards of the American Psychological Association.

#### Apparatus and stimuli

Participants sat comfortably on a chair in front of a 17-inch VGA cathode-ray monitor (refresh rate of 75 Hz, resolution of 1,280 × 1,024) in a dimly lit room, such that their faces were at a distance of roughly 80 cm from the screen. Responses were collected with a USTC Response Time Box (Li, Liang, Kleiner, & Lu, 2010). Microsoft Windows XP served as the operating system and the experiments were written in MATLAB, using the Psychophysics Toolbox extensions (Brainard, 1997; Kleiner et al., 2007; Pelli, 1997).

Prime and target stimuli appeared inside the lollipop frame, which was present throughout the trial (see Fig. 1). The frame was shown in white (54.3 cd/m^2^, line width 2 pixels) against a black background (0.03 cd/m^2^) and consisted of a central circle (Ø 0.8 cm, 0.57°) for the target and a larger circle (Ø 2.4 cm, 1.72°) for the primes. The large circle was subdivided into eight 45° segments by horizontal, vertical and diagonal lines. The first prime (P1) was presented by filling-in four non-contiguous segments with the same color (either red, 11.0 cd/m^2^, *x* = .45, *y* = .30, or green, 11.0 cd/m^2^, *x* = .24, *y* = .40). The second prime (P2) was then presented in the remaining segments. The two sets of segments were randomly assigned to colors and primes. As a target stimulus (T), the inner small circle of the frame was filled with either red or green color.

#### Procedure

Experiment 1 lasted 60 minutes. The experiment started with one practice and two experimental blocks with 50 trials each in which no prime [N] was displayed. This had the purpose to accustom the participants to the procedure. After completion of this task, prime conditions were administered to the participants. Each prime could either be consistent (same color) or inconsistent (different color) with the target. There were two single-prime conditions, consistent [C] and inconsistent [I], and four double-prime conditions, consistent–consistent [CC], consistent–inconsistent [CI], inconsistent–consistent [IC], and inconsistent–inconsistent [II]. (Throughout this paper, we always code consistency relative to the target.) Again, participants had to complete one practice block, this time followed by 25 experimental blocks, with 56 trials each. Each block contained eight single-prime trials and 48 double-prime trials. Altogether, this led to participants completing 100 trials each for the no-prime, two one-prime and twelve double-prime (three SOA × four prime combinations) conditions.^2^

Each trial began with the onset of the lollipop frame (see Fig. 1). After 493 ms of fixation, P1 was presented in either red or green for 13 ms, except for the no-prime trials during which all segments remained black (such that the SOA structure was maintained even when one or both primes were absent). After a P1–P2 SOA of 27, 53, or 80 ms, either a red or green P2 was presented for another 13 ms, except for the no-prime and single-prime trials during which all segments stayed black. Finally, after a P2–T SOA of 80, 53, or 27 ms, a red or green target followed. As a result, the SOA between P1 and T was always 107 ms. The target stayed on-screen for 107 ms. Participants were instructed to fixate the target circle at the center of the frame (see Fig. 1) and to respond to the target color as quickly and accurately by pressing one of two response buttons with their left or right index finger, while all other stimuli were irrelevant. After detection of the manual response, a feedback display was shown for 500 ms, followed by a blank screen for 360 ms before the next trial started. Participants received a “too slow” feedback message if their RT was slower than 999 ms. During practice trials they received an additional “wrong” feedback message if their response was incorrect and “correct” if their response was correct. Additionally, after each block participants received feedback on their performance (percentage correct, number of errors, mean reaction time) and could take a short rest if desired. Color-to-button mapping was fixed for each participant and counterbalanced across participants. All stimulus conditions, except for the blocked no-prime condition, occurred randomly and equiprobably over the course of a session.

#### Analysis of mean error rate and mean correct RT

In a first step, mean reaction times (RT) and error rates (ER) were inspected. We performed two sets of analyses. First, one-way repeated-measures ANOVAs, with the factor consistency (consistent, inconsistent, no prime), were performed for single-prime and no-prime conditions, one for each of the two dependent variables, RT and ER. A total of 3,600 trials were initially available for analysis. Trials with reaction times faster than 100 ms or slower than 999 ms (0.5%) were excluded from the analysis. Further, error trials (10.92%) were excluded from RT analysis.

Second, two 3 (SOA) × 4 (consistency) repeated-measures ANOVAs were performed for all double-prime conditions, one each for RT and ER. A total of 14,400 trials were initially available for analysis. Trials with reaction times faster than 100 ms or slower than 999 ms (0.53%) were excluded from the analysis. In addition, error trials (13.53%) were excluded from RT analysis. To follow up significant interaction effects, one-way repeated-measures ANOVAs, with the four-level factor consistency (CC, CI, IC, II) were performed separately for each SOA condition. Greenhouse–Geisser-corrected *p* values were used. To satisfy ANOVA requirements error rates were arcsine transformed. Additional within-subjects contrasts were calculated to further investigate significant main effects.

#### Event history analysis

Sample-based descriptive estimates of hazard function *h*(t), survival function *S(t)*, probability function *P*(t), and conditional-accuracy function *ca*(t) were calculated for each combination of condition. For the purpose of visually inspecting the descriptive functions data was pooled across participants to reduce noise, after checking that each participant showed similarly timed effects. A censoring time of 600 ms was used because only a limited amount of responses occurred afterwards. To provide a high temporal resolution and still obtain stable estimates a bin size of 25 ms was used. In other words, the first 600 ms after target onset were divided into 24 time bins of 25 ms indexed by t *=* 1 to 24. Trials with RTs longer than 600 ms were treated as right-censored observations. Time bins are denoted by the endpoint of the interval they span, such that Bin 11 = Bin 275 = (250,275].

Next, discrete-time hazard models and conditional accuracy models were estimated by computing linear mixed-effects regression models in *R* (R Core Team, 2014; function glmmPQL^3^ of package MASS; see also Panis & Schmidt, 2016). For the hazard models we used the *complementary log-log (cloglog)* link.^4^ An example discrete-time hazard model with three predictors can be written as follows: cloglog[*h*(t)] = ln(−ln[1 − *h*(t)]) = [α_0_ONE + α_1_(TIME − 1) + α_2_(TIME −1)^2^ + α_3_(TIME − 1)^3^] + [β_1_X_1_ + β_2_X_2_ + β_3_X_2_(TIME − 1)]. The main predictor variable TIME is the time bin index t which is centered on value 1 in this example. The first set of terms within brackets, the alpha parameters multiplied by their polynomial specifications of (centered) time, represents the shape of the baseline cloglog-hazard function (i.e., when all predictors X_i_ take on a value of zero). The second set of terms (the beta parameters) represents the vertical shift in the baseline cloglog-hazard for a 1 unit increase in the respective predictor. For example, the effect of a 1 unit increase in X_1_ is to vertically shift the whole baseline cloglog-hazard function with β_1_ cloglog-hazard units. However, if the predictor interacts linearly with time (see X_2_ in the example), then the effect of a 1 unit increase in X_2_ is to vertically shift the predicted cloglog-hazard in Bin 1 with β_2_ cloglog-hazard units (when TIME − 1 = 0), in Bin 2 with β_2_ + β_3_ cloglog-hazard units (when TIME − 1 = 1), and so forth. To interpret the effects of the predictors, the parameter estimates are antilogged, resulting in a hazard ratio.

For our data we centered TIME on Bin 275 during model selection. TRIAL number was included as a predictor (centered on Trial 1,000, rescaled by dividing by 100), in order to account for across-trial learning effects in *h*(t). The intercept and the linear effect of TIME were treated as random effects to deal with the correlated data resulting from the repeated measures on the same subjects.^5^ The IC-27/80 condition (P1: inconsistent, P2: consistent, SOA1: 27 ms, SOA2: 80 ms) was chosen as a baseline condition. Because TIME and TRIAL are centered, the intercept of the hazard regression model refers to Bin 275 in Trial 1,000 of the IC-27/80 condition.

To estimate the parameters of an *h*(t) model, we must create a dataset where each row corresponds to a time bin of a trial of a participant (a subject-trial-bin oriented data set). Specifically, each time bin that was at risk for event occurrence in a trial was scored on the dependent variable EVENT (0 = *no response occurred*; 1 = *response occurred*), the centered covariates TIME, TRIAL, the variable SUBJECT, and the dummy-coded dichotomous experimental predictor variables (C, I, N, II, CC, CI, SOA_53_53, SOA_80_27). Only the time range between 125 and 450 ms was modeled, because most responses occurred in this range. Trials with RTs longer than 450 ms were treated as right-censored observations, and trials with RT smaller or equal to 125 ms were discarded. The expanded (subject-trial-bin oriented) data set contained 157,656 rows.

For *ca*(t) modeling, the original dataset was used where each row corresponds to one trial of one participant (1,500 × 12 = 18,000 trials). We used the same model but applied the *logit* link^6^, and included only those trials with an observed response between 125 and 450 ms in the data set. In other words, trials with RT shorter than 125 ms and longer than 450 ms were discarded (11.63 % of the 18,000 trials).

For both models, we started with a full model containing all fixed effects of interest (main and interaction effects of the dichotomous predictors), and their interactions with TIME (linear, quadratic, cubic, and quartic). In a step-by-step backward selection procedure, this full model was reduced to the final, selected model. More precisely, in each iteration, the effect with the largest *p* > .05 that was not part of any higher order effect left the model before the next fit. Finally, after model selection, we refitted the selected model a number of times with TIME centered each time on another bin, to see explicitly what values the parameter estimates take on according to the final model in these other bins, and whether they represent a significant effect (see Tables 1 and 2). This way, it becomes more explicit what the interaction effects including TIME imply, because we are able to study the effect of the various predictor variables at different time points.

**Table 1:** Selected hazard model for Experiment 1

		(175,200]		(250,275]				(300,325]		(375,400]	
	Effect	PE	*p*	PE	SE	*t*	*p*	PE	*p*	PE	*p*
1	Intercept	−4.650	0.0000^***^	−2.361	0.260	−9.084	0.0000^***^	−1.318	0.0000^***^	−0.620	0.0000^***^
2	TIME			0.637	0.039	16.504	0.0000^***^				
3	TIME^2^			−0.054	0.004	−14.811	0.0000^***^				
4	TIME^3^			−0.003	0.001	−3.114	0.0018^**^				
5	TIME^4^			0.000	0.000	3.366	0.0008^***^				
6	TRIAL	−0.003	0.5553	0.005	0.003	1.919	0.0549	0.011	0.0000^***^	0.019	0.0000^***^
7	TIME:TRIAL			0.003	0.001	3.418	0.0006^***^				
8	C	0.179	0.1651	0.729	0.055	13.260	0.0000^***^	0.549	0.0000^***^	−0.029	0.6932
9	TIME:C			−0.006	0.025	−0.252	0.8007				
10	TIME^2^:C			−0.050	0.009	−5.615	0.0000^***^				
11	TIME^3^:C			0.004	0.002	2.820	0.0048^**^				
12	I	0.113	0.3951	−0.506	0.070	−7.242	0.0000^***^	−0.688	0.0000^***^	−0.308	0.0000^***^
13	TIME:I			−0.196	0.041	−4.785	0.0000^***^				
14	TIME^2^:I			0.042	0.010	4.291	0.0000^***^				
15	TIME^3^:I			0.008	0.003	2.915	0.0036^**^				
16	TIME^4^:I			−0.001	0.000	−3.736	0.0002^***^				
17	N	0.666	0.0000^***^	0.476	0.061	7.775	0.0000^***^	0.050	0.3661	−0.355	0.0000^***^
18	TIME:N			−0.187	0.024	−7.732	0.0000^***^				
19	TIME^2^:N			−0.024	0.007	−3.373	0.0007^***^				
20	TIME^3^:N			0.006	0.001	4.673	0.0000^***^				
21	II	0.285	0.0012^**^	−0.403	0.054	−7.423	0.0000^***^	−0.623	0.0000^***^	−0.396	0.0000^***^
22	TIME:II			−0.193	0.025	−7.688	0.0000^***^				
23	TIME^2^:II			0.035	0.006	5.714	0.0000^***^				
24	TIME^3^:II			0.005	0.002	3.146	0.0017^**^				
25	TIME^4^:II			−0.001	0.000	−3.747	0.0002^***^				
26	CC	0.389	0.0000^***^	0.627	0.049	12.860	0.0000^***^	0.407	0.0000^***^	−0.084	0.1254
27	TIME:CC			−0.054	0.016	−3.404	0.0007^***^				
28	TIME^2^:CC			−0.034	0.005	−6.597	0.0000^***^				
29	TIME^3^:CC			0.003	0.001	3.835	0.0001^***^				
30	CI	−0.256	0.0000^***^	−0.256	0.032	−8.003	0.0000^***^	−0.256	0.0000^***^	−0.256	0.0000^***^
31	SOA_53_53	−0.191	0.0000^***^	−0.191	0.032	−5.985	0.0000^***^	−0.191	0.0000^***^	−0.191	0.0000^***^
32	SOA_80_27	−0.223	0.0027^**^	−0.327	0.046	−7.053	0.0000^***^	−0.336	0.0000^***^	−0.261	0.0000^***^
33	TIME:SOA_80_27			−0.017	0.012	−1.359	0.1742				
34	TIME^2^:SOA_80_27			0.006	0.002	2.585	0.0097^**^				
35	II:SOA_53_53	0.124	0.0267^*^	0.124	0.056	2.216	0.0267^*^	0.124	0.0267^*^	0.124	0.0267^*^
36	II:SOA_80_27	0.245	0.0001^***^	0.245	0.062	3.928	0.0001^***^	0.245	0.0001^***^	0.245	0.0001^***^
37	CC:SOA_53_53	0.167	0.0021^**^	0.167	0.054	3.078	0.0021^**^	0.167	0.0021^**^	0.167	0.0021^**^
38	CC:SOA_80_27	0.287	0.0000^***^	0.287	0.061	4.693	0.0000^***^	0.287	0.0000^***^	0.287	0.0000^***^
39	CI:SOA_80_27	0.212	0.0001^***^	0.212	0.055	3.845	0.0001^***^	0.212	0.0001^***^	0.212	0.0001^***^
	*SD* Intercept	1.122		.892				.679		.431	
	*SD* TIME	.124		.124				.124		.123	
	Correlation	−.937		−.899				−.818		−.427	

*Note.* Parameter estimates (PE) and test statistics. During model selection, TIME was centered on bin 275. The selected model was refitted three times with TIME centered on bin 200, 325, and 400, respectively. *SD* = standard deviation

**Table 2:** Selected *ca*(t) model for Experiment 1. Parameter estimates (PE) and test statistics

		(150,175]		(200,225]		(250,275]				(375,400]	
	Effect	PE	*p*	PE	*p*	PE	SE	*t*	*p*	PE	*p*
1	Intercept	−2.379	0.0000^***^	−0.076	0.7026	1.918	0.157	12.187	0.0000^***^	3.229	0.0000^***^
2	TIME					0.797	0.062	12.806	0.0000^***^		
3	TIME^2^					−0.118	0.015	−7.696	0.0000^***^		
4	TIME^3^					−0.006	0.003	−1.993	0.0463^*^		
5	TIME^4^					0.002	0.000	4.087	0.0000^***^		
6	C	5.213	0.0000^***^	2.906	0.0000^***^	1.339	0.232	5.764	0.0000^***^	0.659	0.1446
7	TIME:C					−0.599	0.109	−5.516	0.0000^***^		
8	TIME^2^:C					0.093	0.027	3.369	0.0008^***^		
9	I	0.407	0.5240	−2.040	0.0000^***^	−2.405	0.188	−12.783	0.0000^***^	−0.126	0.6165
10	TIME:I					0.188	0.088	2.139	0.0324^*^		
11	TIME^2^:I					0.148	0.025	5.903	0.0000^***^		
12	TIME^3^:I					−0.019	0.004	−4.559	0.0000^***^		
13	N	3.456	0.0000^***^	1.421	0.0000^***^	0.112	0.178	0.629	0.5292	0.013	0.9667
14	TIME:N					−0.473	0.072	−6.584	0.0000^***^		
15	TIME^2^:N					0.091	0.018	5.130	0.0000^***^		
16	II	−0.900	0.1208	−2.376	0.0000^***^	−2.438	0.146	−16.694	0.0000^***^	−0.139	0.4832
17	TIME:II					0.227	0.073	3.108	0.0019^**^		
18	TIME^2^:II					0.106	0.021	4.951	0.0000^***^		
19	TIME^3^:II					−0.012	0.003	−3.655	0.0003^***^		
20	CC	5.349	0.0000^***^	3.189	0.0000^***^	1.624	0.175	9.269	0.0000^***^	0.309	0.2298
21	TIME:CC					−0.634	0.074	−8.596	0.0000^***^		
22	TIME^2^:CC					0.074	0.016	4.659	0.0000^***^		
23	CI	4.742	0.0000^***^	0.487	0.0502	−1.440	0.164	−8.805	0.0000^***^	−0.471	0.0513
24	TIME:CI					−0.494	0.070	−7.041	0.0000^***^		
25	TIME^2^:CI					0.207	0.024	8.478	0.0000^***^		
26	TIME^3^:CI					−0.014	0.004	−3.927	0.0001^***^		
27	SOA_53_53	−0.461	0.0553	−0.391	0.0226^*^	−0.320	0.115	−2.780	0.0054^**^	−0.144	0.3858
28	TIME:SOA_53_53					0.035	0.039	0.893	0.3717		
29	SOA_80_27	−1.177	0.0179^*^	−1.246	0.0000^***^	−1.137	0.168	−6.750	0.0000^***^	−0.091	0.7183
30	TIME:SOA_80_27					0.099	0.075	1.322	0.1862		
31	TIME^2^:SOA_80_27					0.022	0.013	1.736	0.0826		
32	II:SOA_80_27	−0.367	0.6955	0.626	0.1782	1.074	0.234	4.595	0.0000^***^	−0.190	0.5370
33	TIME:II:SOA_80_27					0.088	0.133	0.661	0.5085		
34	TIME^2^:II:SOA_80_27					−0.068	0.023	−2.928	0.0034^**^		
35	CC:SOA_80_27	2.505	0.0002^***^	1.928	0.0001^***^	1.351	0.322	4.199	0.0000^***^	−0.092	0.8261
36	TIME:CC:SOA_80_27					−0.289	0.105	−2.750	0.0060^**^		
37	CI:SOA_53_53	1.327	0.0015^**^	0.957	0.0013^**^	0.587	0.195	3.012	0.0026^**^	−0.339	0.2108
38	TIME:CI:SOA_53_53					−0.185	0.068	−2.733	0.0063^**^		
39	CI:SOA_80_27	4.495	0.0000^***^	3.388	0.0000^***^	2.281	0.256	8.911	0.0000^***^	−0.486	0.1392
40	TIME:CI:SOA_80_27					−0.553	0.085	−6.521	0.0000^***^		
	*SD* Intercept	.373		.324		.353				.353	
	*SD* TIME	.081		.081		.081				.081	
	Correlation	−.496		−.068		.398				.406	

*Note.* During model selection TIME was centered on bin 275. The selected model was refitted three times with TIME centered on bin 175, 225, and 400, respectively

#### Predictions

We expected primes to have sequential effects that are traceable over time in the conditional accuracy functions. Because P1–T SOAs in Experiment 1 are short, the sequence of response activations should conform to the chase criteria, so that the earliest responses are controlled exclusively by the first prime, while later responses are consecutively controlled by the second prime, and the slowest responses by the target. The earliest responses should therefore be correct whenever P1 is consistent with the target and incorrect whenever it is inconsistent. In contrast, the slowest responses should be controlled mainly by the target and thus all be correct. Intermediate responses should be influenced by the second prime. From previous data, we expected that the second prime would dominate the response at the shortest P1–P2 SOA (i.e., the longest P2–T SOA), and this dominance of the second prime should decrease with increasing P1–P2 SOA because the first prime has progressively more time to activate a response before the second prime occurs, while the second prime has progressively less time before the target occurs (Grainger et al., 2013).

### Results

#### Analysis of mean error rate and mean correct RT

**Fig. 2:**
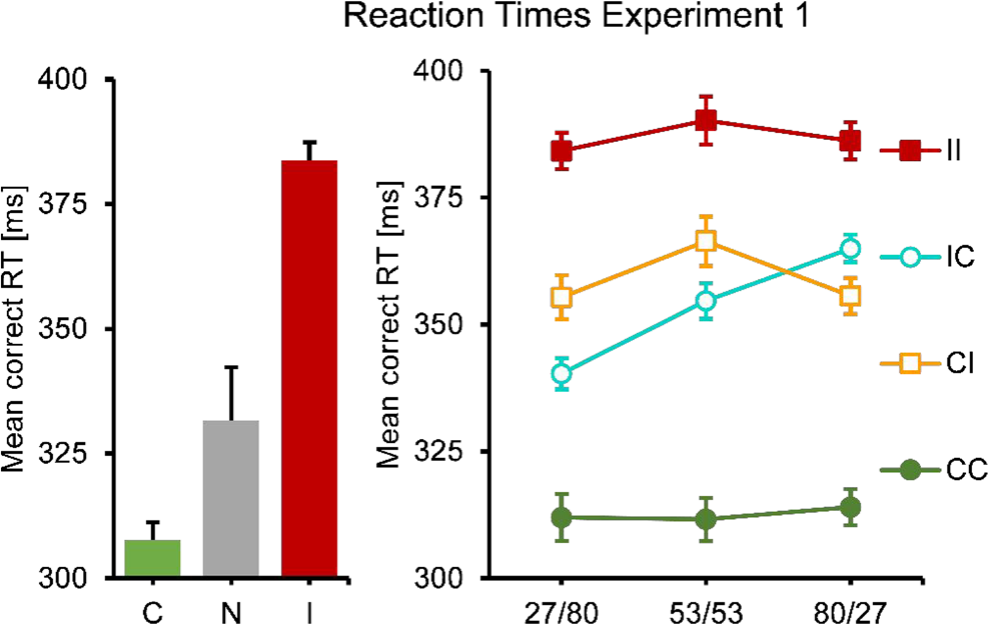
Mean correct RT results for Experiment 1. No and single-prime conditions: Left panel, error bars resemble the standard error of the mean, consistency conditions on the *x*-axes. Double-prime conditions: Right panel, error bars resemble the standard error of the mean, separate lines for consistency conditions, SOA conditions on the *x*-axes

**Fig. 3:**
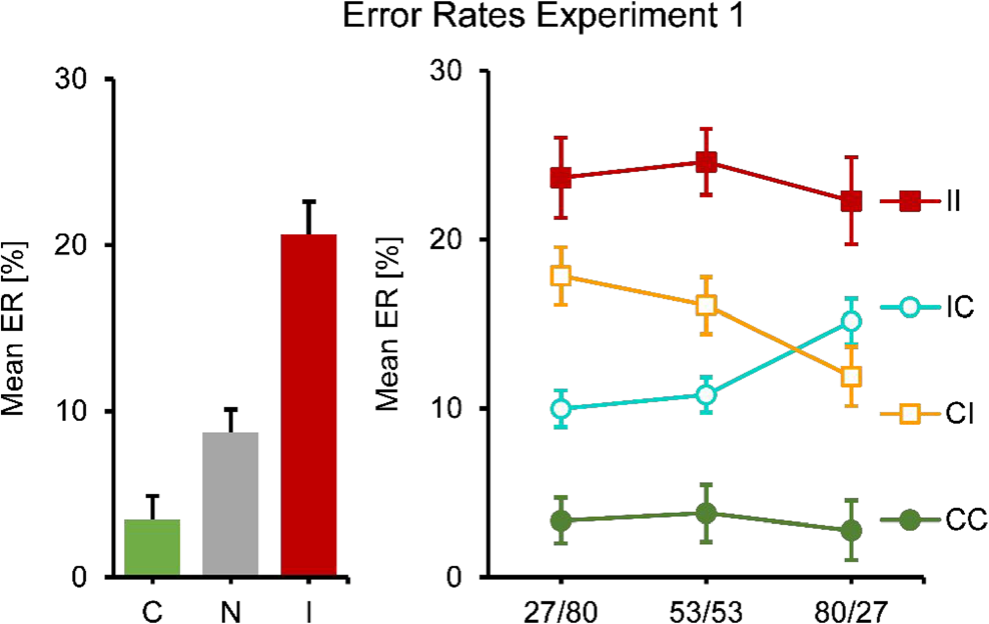
Mean ER results for Experiment 1. No and single-prime conditions: Left panel, error bars resemble the standard error of the mean, consistency conditions on the *x*-axes. Double-prime conditions: Right panel, error bars resemble the standard error of the mean, separate lines for consistency conditions, SOA conditions on the *x*-axes

An analysis of the single prime conditions showed that responses were faster and more accurate when primes were consistent rather than inconsistent, with the no-prime condition in between (see Figs. 2 and 3, left panel). One-way repeated-measures ANOVAs showed significant differences in RT, *F*(1.45, 15.90) = 33.39, *p* < .001, as well as error rates, *F*(1.84, 20.25) = 35.56, *p* < .001. In RTs as well as error rates, all means were significantly different from each other, all *p* ≤ .001, except for the RT difference between consistent and no-prime conditions (*p* = .061).

In a next step, double-prime conditions were analyzed. RTs and error rates showed a similar overall pattern: Responses were fastest and most accurate for two consistent primes, slowest and least accurate for two inconsistent primes, and in between when primes were mixed (conditions CI and IC). In RTs, a two-way repeated-measures ANOVA showed a significant main effect of consistency (with levels CC, CI, IC, II), *F*(2.09, 22.98) = 64.94, p < .001, a significant main effect of SOA *F*(1.85, 20.35) = 10.43, *p* = .001, and a significant interaction, *F*(4.00, 44.04) = 7.86, *p* < .001 (see Fig. 2, right panel). This pattern was broken down into two separate ANOVAs, one for identical (CC, II) and one for different primes (CI, IC). The first one (CC versus II) only showed a significant main effect of consistency, *F*(1.00, 11.00) = 113.30, *p* < .001. This effect was constant across SOA, with no main effect of SOA or an interaction. The second test (CI versus IC) showed that RT increased with SOA, *F*(1.78, 19.32) = 27.76, *p* < .001. There was no main effect of consistency, but a significant interaction, *F*(1.46, 16.10) = 12.69, *p* = .001. IC was faster than CI when the first SOA was short, but slower when it was long.

This pattern was even clearer (and almost perfectly symmetrical) in the error rates. An ANOVA of all double-prime conditions showed no main effect of SOA, but a significant main effect of consistency, *F*(1.73, 19.07) = 37.84, *p* < .001, and a significant interaction *F*(3.38, 37.18) = 5.61, *p* = .002. The follow-up analysis of CC versus II conditions only showed a main effect of consistency, *F*(1.00, 11.00) = 59.40, *p* < .001, but no SOA or interaction effects. The follow-up analysis of CI versus IC conditions only showed an interaction, *F*(1.98, 21.74) = 13.87, *p* < .001, but no main effects: IC was more accurate than CI when the first SOA was short, but less accurate when it was long.

#### Event history analysis: Descriptive statistics

**Fig. 4:**
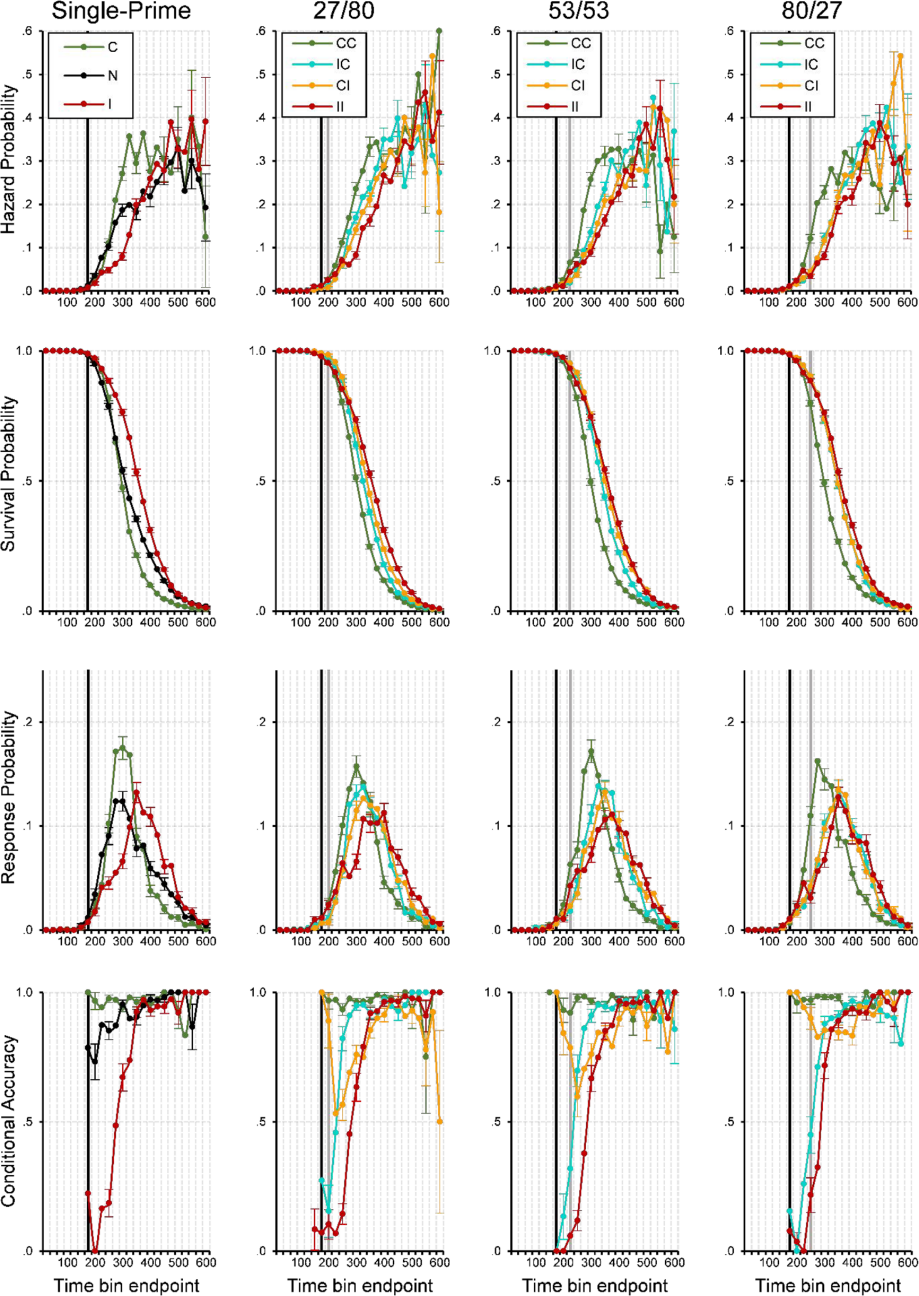
Sample-based estimates of *h*(t), *S*(t), *P*(t), and *ca*(t) aggregated across all participants in Experiment 1, for the first 24 bins (or 600 ms) after target onset. Bin width equals 25 ms. First column: Black lines represent the no-prime condition, green lines the consistent single-prime condition, and red lines the inconsistent single-prime condition. Second to last column: Each column represents a different SOA condition. Green lines represent consistent–consistent conditions, cyan lines inconsistent–consistent conditions, orange lines consistent–inconsistent conditions, red lines inconsistent–inconsistent conditions. Black vertical lines highlight bins at ~250–275 ms after onset of P1, grey vertical lines after onset of P2. Note that we only plotted a *ca*(t) estimate if the corresponding hazard for that bin was larger than .005. For better visibility only every second error bar is depicted

In the single-prime conditions (see the first column in Fig. 4), the fastest responses occurred around 150 ms after target onset. Thereafter, we saw a steady increase in response hazards, which was delayed for inconsistent compared with consistent primes. This led to a marked priming effect in *h*(t) of about 150 ms duration, and also in median RT (i.e., when the survivor function crosses .5) and mean RT. When most responses had occurred and the survival probability was low, response hazard was still at a high constant level. Strikingly, early responses were virtually always correct whenever the prime was consistent, but incorrect whenever it was inconsistent, showing that responses were exclusively determined by the prime, not the target. In inconsistent trials, conditional accuracy then quickly increased from almost zero to almost one, showing how the target took control over the response.^7^

Let us now take a look at the double-prime conditions where the P1–P2 SOA was short and the P2–T SOA was long (27/80’ see the second column in Fig. 4), so that the impact of the second prime should be high relative to the first prime. Again, the fastest responses occurred around the same time in all priming conditions, around 150 ms after target onset or about 250 ms after P1 onset. However, initial response hazards in CI and IC conditions were lower than in CC and II conditions around 150–200 ms. This likely reflected early response competition due to conflicting prime information, as both primes activated opposite responses. After about 250 ms without response occurrence, the hazard functions began to differentiate and followed the order observed in mean RTs: CC was fastest, followed by IC, CI, II. This was evident in the hazard, survivor, and probability mass functions. The most diagnostic information, however, was in the conditional accuracy functions. Not surprisingly, the earliest responses were virtually all correct when both primes were consistent and all incorrect when both primes were inconsistent, which again showed that the first prime determined the earliest responses. In the II condition, conditional accuracy then quickly increased as the target took control over the response. This also occurred in the IC condition, demonstrating that the first prime alone controlled the earliest responses; but the following increase in accuracy occurred earlier than in the II condition, demonstrating that the consistent second prime influenced the response as well. Exactly the reverse process occurred in the CI condition. Here, response accuracy was nearly perfect at first because of the consistent first prime, then decreased as the inconsistent second prime became effective, and then increased again as the target finally took control over the response.

What happened when the SOAs shifted first to 53/53 and then to 80/27? The overall pattern in *ca*(t) remained the same: CC was always fastest with near-perfect accuracy, II was always slowest with conditional accuracy rising from very low to very high values, IC always showed an earlier increase in conditional accuracy, time-locked to the second prime’s appearance. The most important change occurred in the CI condition. As the P1–P2 SOA became larger and the P2–T SOA became correspondingly shorter, the influence of the inconsistent second prime diminished, and the temporary drop in conditional accuracy became smaller. As with the effects observed in IC, this nadir in conditional accuracy was time locked to the second prime’s presentation.

#### Event history analysis: Inferential statistics

**Fig. 5:**
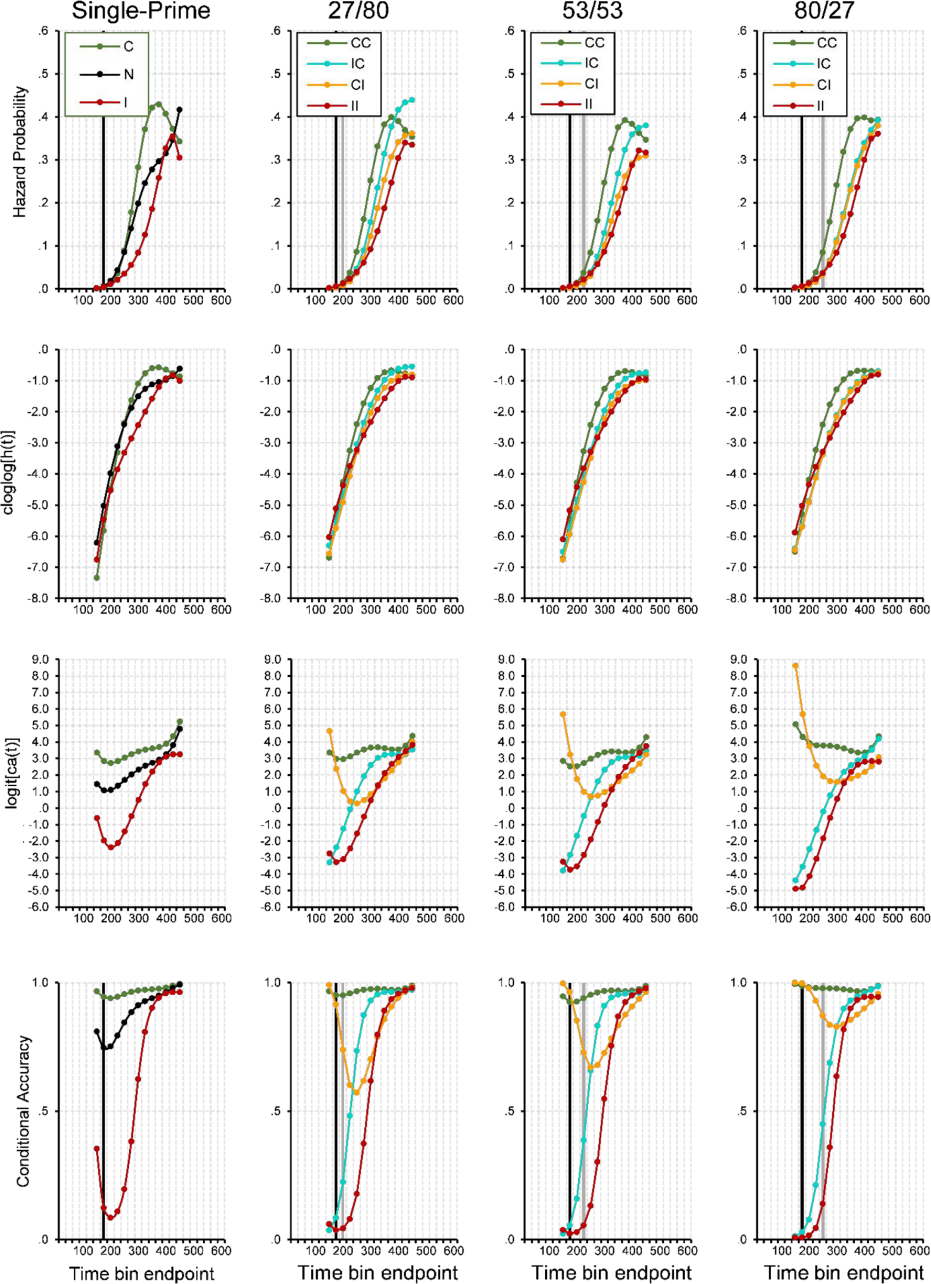
Model predictions. Predicted hazard (first row), cloglog[*h*(t)] (second row), logit[*ca*(t)] (third row), and conditional accuracy functions (fourth row) for trial 1,000 of Experiment 1. Again, black vertical lines highlight bins at ~250–275 ms after onset of P1, grey vertical lines after onset of P2

To see whether these observed differences are significant we fitted hazard and conditional accuracy models to the aggregated data. Table 1 shows the selected hazard model, and Table 2 the selected *ca*(t) model. Figure 5 shows predicted (i.e., model-based) hazard cloglog[*h*(t)], logit[*ca*(t)], and conditional accuracy functions for Trial 1,000 (note that choosing another trial number would not change the priming effects because we did not include interaction effects including TRIAL). The first five parameters in Table 1 model the shape of the cloglog[*h*(t)] function in the baseline condition, IC-27/80 in Trial 1,000 (see Fig. 5, row 2, column 2, blue line). The intercept of −2.361 cloglog-hazard units corresponds to an estimated hazard of .09 in Bin 275. This intercept increases over time in a linear, quadratic, cubic and quartic fashion (see the Parameters 2 to 5 in Table 1), so that the intercept changes from −4.65 in Bin 200 to −.62 in Bin 400 (see row 1 in Table 1).

Most importantly, compared with condition IC, changing to CC increases the estimated cloglog-hazard in Bin 275 by .627 units (Parameter 26), changing to CI decreases it by .256 units (Parameter 30), and changing to II decreases it by .403 units (Parameter 21; all *p* < .0001). While the main effects of CC and II in Bin 275 change in magnitude over time (parameter estimates in rows 27–29, 22–25), the effect of CI is time invariant. For example, note that in Bin 200 conditions, II and CC have positive parameter estimates that significantly differ from condition IC (see the parameter estimates in rows 21 and 26 in Table 1, column 3). This means that the hazard of response occurrence is lower in Bin 200 in mixed prime conditions.

The effect of changing the SOA combination from 27/80 to 53/53 is to decrease the estimated cloglog-hazard by .191 units in all bins (Parameter 31). The estimated cloglog-hazard decreases even further when SOA combination is changed to 80/27 (Parameters 32–34). In other words, response occurrence slows down with decreasing P2–T SOA for condition IC. However, this effect is much smaller or absent for CC and II due to interactions with 53/53 (Parameters 35 and 37) and 80/27 (Parameters 36 and 38). Furthermore, with SOA combination 80/27 the difference between CI and IC is gone (due to Parameter 39 neutralizing the effect of Parameter 30). Finally, the hazard model also shows a significant effect of TRIAL on the estimated cloglog-hazard in bins after 275 ms after target onset.

The first five parameters in Table 2 model the shape of the logit[*ca*(t)] function in the baseline condition, IC-27/80 in Trial 1,000 (see Fig. 5, row 3, column 2, blue line). The intercept of 1.918 corresponds to an estimated *ca*(t) of .87 in Bin 275. This intercept increases over time in a linear, quadratic, cubic and quartic fashion (Parameters 2–5).

Most importantly, compared with condition IC, changing to CC increases the estimated logit-*ca*(t) in Bin 275 by 1.624 units (Parameter 20), changing to CI decreases it by 1.44 units (Parameter 23), and changing to II decreases it by 2.438 units (Parameter 16; all *p*s < .0001). The main effects of CC, CI, and II in Bin 275 change over time (Parameters 16–26), so that relative to IC, the positive effect of CC decreases over time, the negative effect of II first increases and then decreases, and the effect of CI shifts from positive to negative to zero. For example, note that in Bin 175 conditions CC and CI have positive parameter estimates that significantly differ from condition IC while II is not significantly different (compare rows 20 and 23 with row 16 in Table 2, column 3). This means that the conditional accuracy of these early responses is almost zero for II and IC, and almost one for CI and CC, thus reflecting first prime identity (see Fig. 5, row 4).

Increasing the P1–P2 SOA leads to a decrease in the estimated logit-*ca*(t) in each bin (Parameters 27–31). Confirming the change in the temporary drop in conditional accuracy for condition CI in Fig. 4 are the (early and positive) interactions between CI, SOA combination, and TIME (Parameters 37–40).

#### Summary

As expected, mean RT and mean ER analyses of the single-prime and no-prime conditions revealed that the stimulus-set used was sufficient to produce the common finding in response priming experiments: faster and more accurate responses in consistent trials and slower and less accurate responses in inconsistent trials. Similarly, when two primes were presented, responses were fastest and most accurate for two consistent primes, slowest and least accurate for two inconsistent primes, and in between when primes were mixed. The event history analysis showed that sequential primes in fact initiate sequential response activation: (1) earliest *responses* were controlled exclusively by the first prime, (2) intermediate responses reflected competition between the primes where the identity of the second prime increasingly dominated the response as P2–T SOA increased, (3) this latter effect was tracking the onset of the second prime, both in magnitude and timing, and (4) only the slowest responses were clearly controlled by the target.

## Experiment 2

### Method

#### Participants

All participants from the first experiment also took part in the second experiment (see Participants section for Experiment 1). Experiment order was counterbalanced.

#### Apparatus and stimuli

The same apparatus and stimuli were employed (see Apparatus and Stimuli section in Experiment 1).

#### Procedure

**Fig. 6:**
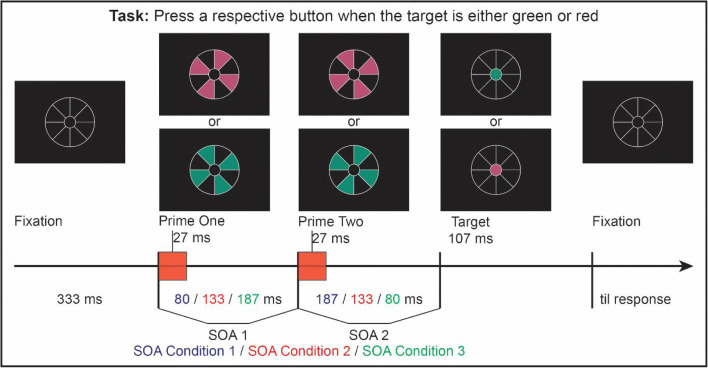
Stimulus displays and design. After fixating the center of the white lollipop frame a sequence of two primes and a target is presented, with SOA1–SOA2 combinations of 80/187, 133/133, or 187/80

Again, each trial began with the onset of the lollipop frame (see Fig. 6). This time, after 333 ms of fixation, P1 was presented in either red or green for 27 ms, except for the no-prime trials, during which all segments remained black. After a P1–P2 SOA of 80, 133, or 187 ms, either a red or green P2 was presented for 27 ms, except for the no-prime and single-prime trials, during which all segments remained black. After a P2–T SOA of 187, 133, or 80 ms, respectively, a red or green target was presented. In this experiment, P1–P2 and P2–T SOAs always added up to a P1–T SOA of 267 ms. The target stayed on-screen for 107 ms.

#### Analysis of mean error rate and mean correct RT

In a first step, two sets of analyses were performed. First, one-way repeated-measures ANOVAs, with the factor consistency (consistent, inconsistent, no prime), were performed for single-prime and no-prime conditions, one for each of the two dependent variables, RT and ER. A total of 3,600 trials were initially available for analysis. Trials with reaction times faster than 100 ms or slower than 999 ms (0.58%) were excluded from the analysis. Further, error trials (then 8.16%) were excluded from RT analysis.

Second, two 3 (SOA) × 4 (consistency) repeated-measures ANOVAs were performed for all double-prime conditions, one each for RT and ER. Initially, a total of 14,400 trials were available for analysis. Trials with reaction times faster than 100 ms or slower than 999 ms (0.93%) were excluded from the analysis. Further, error trials (then 12.88%) were excluded from RT analysis. To follow up on significant interaction effects, one-way repeated-measures ANOVAs, with the four-level factor consistency, were performed separately for each SOA condition.

Greenhouse–Geisser-corrected *p* values were used. To satisfy ANOVA requirements, error rates were arcsine transformed. Additional within-subjects contrasts were calculated to further investigate significant main effects.

#### Event history analysis

First, descriptive statistics were calculated as in Experiment 1 (see Event History Analysis section in Experiment 1). Next, for hazard modeling purposes, we censored the trials at 450 ms after target onset, and discarded the first five bins, since the most informative events occurred within 125 to 450 ms. The final data set for fitting *h*(t) models contained 153,286 rows.

Finally, for *ca*(t) modeling, the original data set was used where each row corresponds to one trial of one participant (1,500 × 12 = 18,000 trials). Trials with a response latency below 125 ms or above 450 ms were deleted (12.36% of the data), in order to avoid problems of linear separability during model fitting. The final data set for the *ca*(t) model contained 15,775 rows.

The estimation procedures were the same for both models as in Experiment 1, except that now the IC-187/80 condition (P1: inconsistent, P2: consistent, P1–P2 SOA: 187 ms, P2–T SOA: 80 ms) was chosen as a baseline condition. In summary, with all effects set to zero, the *h*(t) model’s intercept refers to the estimated cloglog[*h*(t)], and the *ca*(t) model’s intercept to the estimated logit[*ca*(t)], for bin 275 in Trial 1,000 of the IC-187/80 condition. Again, we refitted the selected model a number of times, with TIME centered each time on another bin (see Tables 3 and 4).

**Table 3:** Selected hazard model for Experiment 2

		(150,175]		(250,275]				(300,325]		(375,400]	
	effect	PE	*p*	PE	SE	*t*	*p*	PE	*p*	PE	*p*
1	Intercept	−5.941	0.0000^***^	−2.162	0.259	−8.355	0.0000^***^	−1.190	0.0000^***^	−0.843	0.0000^***^
2	TIME			0.666	0.037	18.245	0.0000^***^				
3	TIME^2^			−0.089	0.004	−23.066	0.0000^***^				
4	TIME^3^			−0.002	0.001	−2.467	0.0136^*^				
5	TIME^4^			0.001	0.000	6.131	0.0000^***^				
6	TRIAL	0.008	0.0000^***^	0.008	0.002	4.099	0.0000^***^	0.008	0.0000^***^	0.008	0.0000^***^
7	C	0.128	0.4836	0.315	0.050	6.343	0.0000^***^	0.268	0.0000^***^	0.022	0.7489
8	TIME:C			0.000	0.026	−0.001	0.9995				
9	TIME^2^:C			−0.012	0.005	−2.337	0.0194^*^				
10	I	−0.279	0.1733	−0.442	0.056	−7.876	0.0000^***^	−0.383	0.0000^***^	−0.118	0.0377^*^
11	TIME:I			0.006	0.029	0.217	0.8282				
12	TIME^2^:I			0.012	0.005	2.409	0.0160^*^				
13	N	1.282	0.0000^***^	0.337	0.053	6.368	0.0000^***^	0.034	0.5100	−0.208	0.0024^**^
14	TIME:N			−0.180	0.022	−8.171	0.0000^***^				
15	TIME^2^:N			0.014	0.004	3.248	0.0012^**^				
16	II	1.188	0.0000^***^	−0.569	0.059	−9.696	0.0000^***^	−0.790	0.0000^***^	−0.394	0.0000^***^
17	TIME:II			−0.236	0.028	−8.398	0.0000^***^				
18	TIME^2^:II			0.063	0.006	10.072	0.0000^***^				
19	TIME^3^:II			0.001	0.001	0.662	0.5082				
20	TIME^4^:II			−0.001	0.000	−2.521	0.0117^*^				
21	CC	0.754	0.0000^***^	0.335	0.038	8.733	0.0000^***^	0.194	0.0000^***^	0.069	0.1961
22	TIME:CC			−0.082	0.019	−4.397	0.0000^***^				
23	TIME^2^:CC			0.006	0.004	1.522	0.1281				
24	CI	−0.100	0.5469	−0.529	0.047	−11.279	0.0000^***^	−0.599	0.0000^***^	−0.358	0.0000^***^
25	TIME:CI			−0.095	0.027	−3.494	0.0005^***^				
26	TIME^2^:CI			0.026	0.006	4.155	0.0000^***^				
27	TIME^3^:CI			0.003	0.002	1.769	0.0769				
28	TIME^4^:CI			−0.001	0.000	−2.630	0.0085^**^				
29	SOA_80_187	0.780	0.0000^***^	0.350	0.049	7.174	0.0000^***^	0.200	0.0000^***^	0.056	0.3904
30	TIME:SOA_80_187			−0.086	0.020	−4.252	0.0000^***^				
31	TIME^2^:SOA_80_187			0.005	0.003	1.621	0.1050				
32	SOA_133_133	0.275	0.0000^***^	0.160	0.032	5.045	0.0000^***^	0.103	0.0000^***^	0.018	0.6524
33	TIME:SOA_133_133			−0.029	0.010	−2.884	0.0039^**^				
34	II:SOA_80_187	−0.933	0.0000^***^	−0.080	0.078	−1.025	0.3055	0.137	0.0550	0.202	0.0222^*^
35	TIME:II:SOA_80_187			0.143	0.030	4.809	0.0000^***^				
36	TIME^2^:II:SOA_80_187			−0.017	0.005	−3.336	0.0008^***^				
37	II:SOA_133_133	−0.381	0.0028^**^	−0.169	0.068	−2.496	0.0126^*^	−0.063	0.2253	0.096	0.1665
38	TIME:II:SOA_133_133			0.053	0.018	2.925	0.0034^**^				
39	CC:SOA_80_187	−0.818	0.0000^***^	−0.127	0.064	−1.982	0.0474^*^	−0.007	0.9181	−0.107	0.2798
40	TIME:CC:SOA_80_187			0.098	0.029	3.364	0.0008^***^				
41	TIME^2^:CC:SOA_80_187			−0.019	0.006	−3.154	0.0016^**^				
42	CI:SOA_80_187	−0.268	0.0534	−0.093	0.069	−1.337	0.1811	−0.005	0.9291	0.127	0.1213
43	TIME:CI:SOA_80_187			0.044	0.021	2.082	0.0374^*^				
	*SD* Intercept	1.301		.888				.696		.460	
	*SD* TIME	.111		.111				.111		.111	
	Correlation	−.954		−.898				−.828		−.528	

*Note.* Parameter estimates (PE) and test statistics. During model selection TIME was centered on bin 275. The selected model was refitted three times with TIME centered on bin 175, 325, and 400, respectively

**Table 4:** Selected *ca*(t) model for Experiment 2

		(175,200]		(250,275]				(300,325]		(375,400]	
	Effect	PE	*p*	PE	SE	*t*	*p*	PE	*p*	PE	*p*
1	Intercept	1.861	0.0000^***^	2.827	0.176	16.059	0.0000^***^	3.139	0.0000^***^	3.603	0.0000^***^
2	TIME			0.188	0.054	3.499	0.0005^***^				
3	TIME^2^			−0.025	0.013	−1.968	0.0491^**^				
4	TIME^3^			0.005	0.003	1.877	0.0605				
5	TIME^4^			0.000	0.000	−0.784	0.4328				
6	C	0.700	0.0378^*^	0.223	0.207	1.079	0.2807	−0.095	0.5991	−0.573	0.0359^*^
7	TIME:C			−0.159	0.061	−2.599	0.0094^**^				
8	I	−3.641	0.0000^***^	−1.287	0.193	−6.660	0.0000^***^	−0.322	0.1310	0.218	0.4510
9	TIME:I			0.603	0.095	6.345	0.0000^***^				
10	TIME^2^:I			−0.060	0.020	−2.998	0.0027^**^				
11	N	−0.723	0.0000^***^	−0.723	0.158	−4.584	0.0000^***^	−0.723	0.0000^***^	−0.723	0.0000^***^
12	II	−6.031	0.0000^***^	−2.997	0.173	−17.332	0.0000^***^	−1.590	0.0000^***^	−0.402	0.0576
13	TIME:II			0.827	0.064	12.927	0.0000^***^				
14	TIME^2^:II			−0.062	0.013	−4.679	0.0000^***^				
15	CC	0.961	0.0011^**^	0.111	0.151	0.731	0.4645	0.229	0.1297	0.313	0.1589
16	TIME:CC			−0.022	0.073	−0.298	0.7660				
17	TIME^2^:CC			0.059	0.019	3.039	0.0024^**^				
18	TIME^3^:CC			−0.009	0.004	−2.637	0.0084^**^				
19	CI	−3.660	0.0000^***^	−3.678	0.188	−19.545	0.0000^***^	−2.233	0.0000^***^	−1.129	0.0000^***^
20	TIME:CI			0.721	0.103	7.017	0.0000^***^				
21	TIME^2^:CI			0.072	0.025	2.823	0.0048^**^				
22	TIME^3^:CI			−0.044	0.006	−6.856	0.0000^***^				
23	TIME^4^:CI			0.004	0.001	4.837	0.0000^***^				
24	SOA_80_187	0.972	0.0034^**^	−0.013	0.170	−0.076	0.9396	−0.386	0.0178^*^	−0.574	0.0081^**^
25	TIME:SOA_80_187			−0.240	0.065	−3.706	0.0002^***^				
26	TIME^2^:SOA_80_187			0.028	0.022	1.291	0.1967				
27	TIME^3^:SOA_80_187			0.000	0.004	−0.128	0.8984				
28	SOA_133_133	−0.097	0.7321	0.051	0.162	0.317	0.7513	0.034	0.8416	−0.166	0.4399
29	TIME:SOA_133_133			0.015	0.056	0.260	0.7949				
30	TIME^2^:SOA_133_133			−0.012	0.012	−0.978	0.3280				
31	II:SOA_80_187	1.084	0.0000^***^	1.084	0.207	5.239	0.0000^***^	1.084	0.0000^***^	1.084	0.0000^***^
32	II:SOA_133_133	0.724	0.0008^***^	0.724	0.215	3.365	0.0008^***^	0.724	0.0008^***^	0.724	0.0008^***^
33	CI:SOA_80_187	−1.783	0.0058^**^	1.598	0.254	6.289	0.0000^***^	1.640	0.0000^***^	0.281	0.3841
34	TIME:CI:SOA_80_187			0.369	0.119	3.089	0.0020^**^				
35	TIME^2^:CI:SOA_80_187			−0.205	0.040	−5.113	0.0000^***^				
36	TIME^3^:CI:SOA_80_187			0.016	0.006	2.538	0.0112^*^				
37	CI:SOA_133_133	−0.146	0.7966	0.672	0.230	2.922	0.0035^**^	0.769	0.0010^**^	0.242	0.4493
38	TIME:CI:SOA_133_133			0.138	0.111	1.242	0.2142				
39	TIME^2^:CI:SOA_133_133			−0.045	0.023	−1.987	0.0470^*^				
	*SD* Intercept	.231		.390				.496		.655	
	*SD* TIME	.053		.053				.053		.053	
	Correlation	.988		.996				.998		.999	

*Note*. Parameter estimates (PE) and test statistics. During model selection TIME was centered on bin 275. The selected model was refitted three times with TIME centered on bin 200, 325, and 400, respectively

#### Predictions

Because P1–T SOAs in Experiment 2 are long, responses are no longer expected to conform to the chase criteria because participants have to wait out the target in order to safeguard against errors provoked by the primes, so that early primes can influence responses only out of the memory buffer that carries information from both primes but is dominated by the second one (Grainger et al., 2013). Therefore, we expected that early responses would no longer be controlled exclusively by the first prime, but jointly by both primes, with the second prime becoming more dominant as the P2–T SOA increased. The latest responses should be controlled mainly by the target and thus all be correct.

### Results

#### Analysis of mean error rate and mean correct RT

**Fig. 7:**
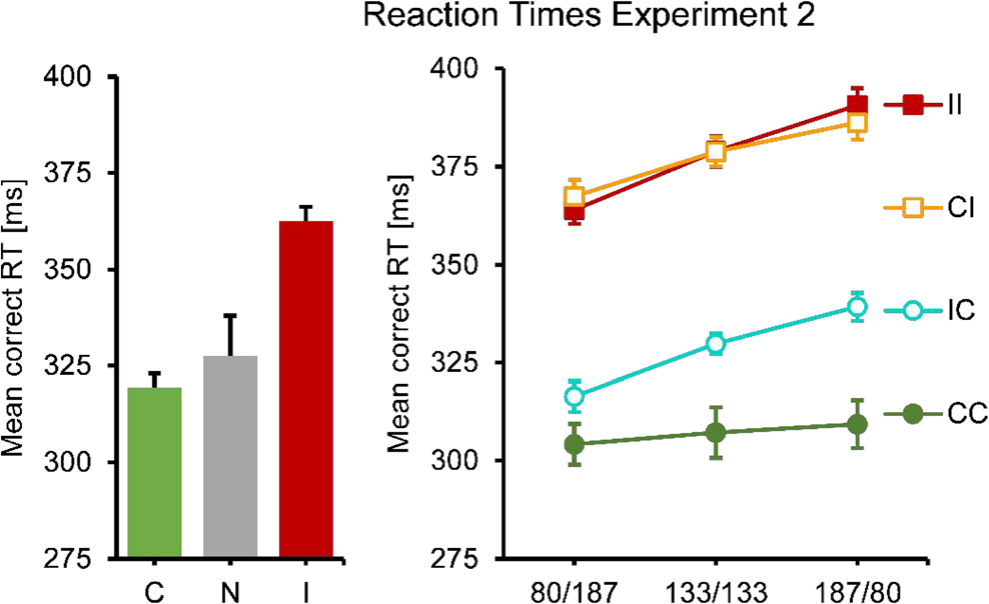
Mean correct RT results for Experiment 2. No and single-prime conditions: Left panel, error bars resemble the standard error of the mean, consistency conditions on the *x*-axes. Double-prime conditions: Right panel, error bars resemble the standard error of the mean, separate lines for consistency conditions, SOA conditions on the *x*-axes

**Fig. 8:**
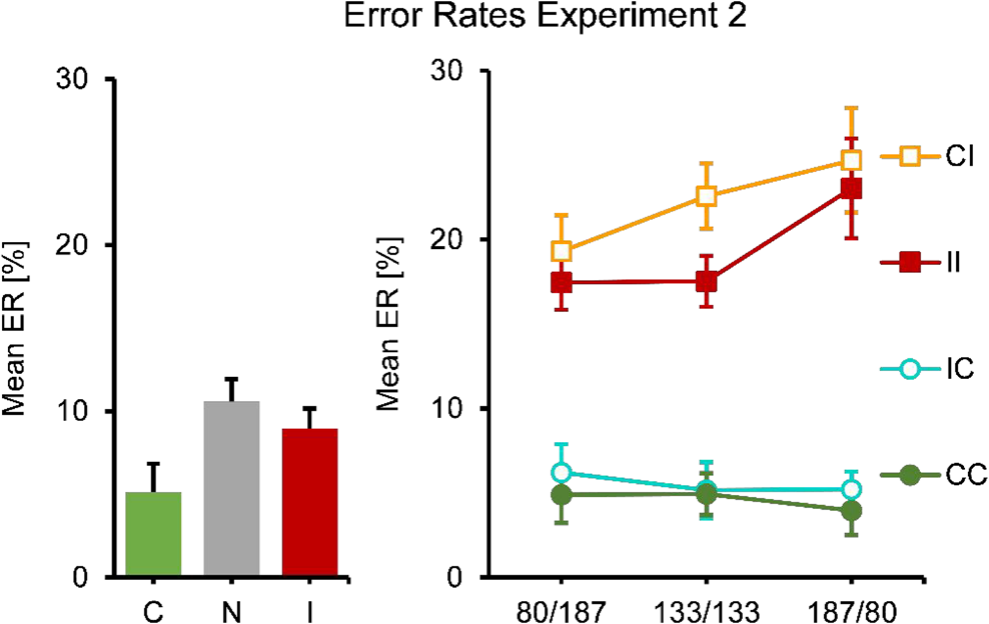
Mean ER results for Experiment 2. No and single-prime conditions: Left panel, error bars resemble the standard error of the mean, consistency conditions on the *x*-axes. Double-prime conditions: Right panel, error bars resemble the standard error of the mean, separate lines for consistency conditions, SOA conditions on the *x*-axes

Analysis of the single prime conditions showed that responses were faster and more accurate when primes were consistent rather than inconsistent. The no-prime condition was intermediate in response times, but higher than the other two in error rate (see Figs. 7 and 8, left panel). One-way repeated-measures ANOVAs showed significant differences in RT, *F*(1.34, 14.75) = 12.60, *p* = .002, as well as error rates, *F*(1.82, 20.02) = 3.95, *p* = .039. In RTs, the differences between all means were significant, all *p*s ≤ .002, except the one between consistent and no primes. In ER, only the difference between consistent and no primes was significant, *p* = .035.

In a next step, double-prime conditions were analyzed (see Figs. 7 and 8, right panel). Responses were fastest and most accurate for two consistent primes and slowest and less accurate for two inconsistent primes. The CI condition was virtually identical to the II condition in response times, but slightly higher in error rate. The IC condition was similar to the CC condition in error rates, but slower in terms of response times. In RTs, a two-way repeated-measures ANOVA showed a significant main effect of consistency (with levels CC, CI, IC, II), *F*(1.55, 17.00) = 59.88, *p* < .001, a significant main effect of SOA, *F*(1.62, 17.83) = 87.63, *p* < .001, and a significant interaction, *F*(3.35, 36.88) = 3.66, *p* = .018, that seems to be based on the less steep increase in RT with SOA in the CC condition. We broke down this pattern post hoc into two separate ANOVAs, one for inconsistent and one for consistent second primes. The first one (II versus CI) only showed that response time increased with SOA, *F*(1.73, 19.01) = 87.91, *p* < .001. The second test (CC versus IC) showed that responses were faster for CC than for IC, *F*(1.00, 11.00) = 15.44, *p* = .002, that RT increased with SOA, *F*(1.66, 18.30) = 15.66, *p* < .001, and that the increase was steeper for IC than for CC, *F*(1.68, 18.46) = 6.11, *p* = .012.

The same strategy was used for the error rates. An ANOVA of all dual-prime conditions showed no main effect of SOA, but a significant main effect of consistency, *F*(1.83, 20.08) = 45.15, *p* < .001, and an interaction effect, *F*(4.26, 46.82) = 2.55, *p* = .048. The analysis of CC versus IC conditions gave no significant effects, and neither did the analysis of II versus CI conditions.

#### Event history analysis: Descriptive statistics

**Fig. 9:**
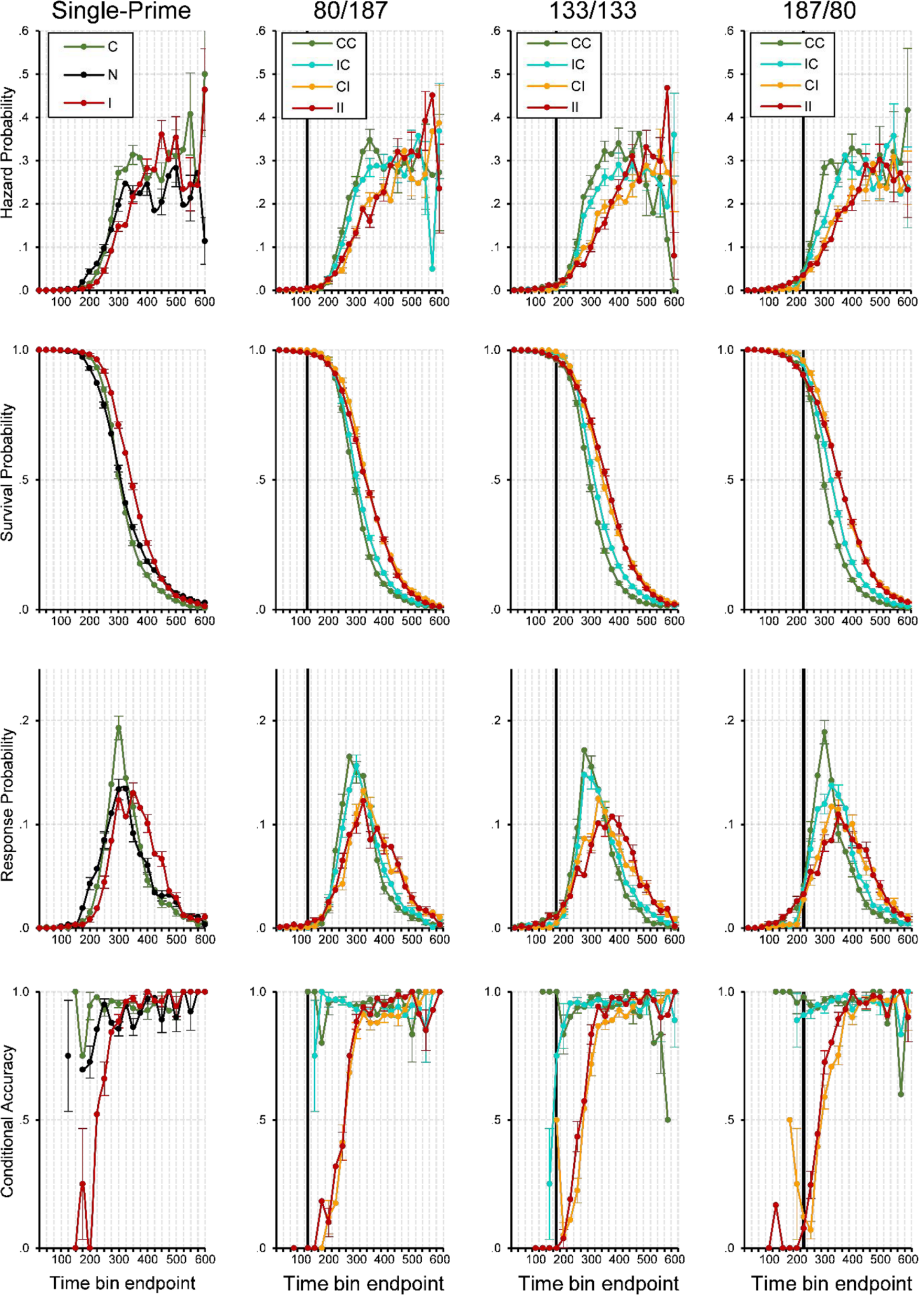
Sample-based estimates of *h*(t), *S*(t), *P*(t), and *ca*(t) aggregated across all participants in Experiment 2, for the first 24 bins (or 600 ms) after target onset. Bin width equals 25 ms. (First column) Black lines represent the no-prime condition, green lines the consistent single-prime condition, and red lines the inconsistent single-prime condition. (Second to last column) Each column represents a different SOA condition. Green lines represent consistent-consistent conditions, cyan lines inconsistent-consistent conditions, orange lines consistent-inconsistent conditions, red lines inconsistent–inconsistent conditions. Black vertical lines highlight bins at ~275–300 ms after onset of P2. Note that we only plotted a *ca*(t) estimate if the corresponding hazard for that bin was larger than .003. For better visibility only every second error bar is depicted

In the single-prime conditions (first column in Fig. 9), the first responses occur after about 200 ms, which is a bit later than in Experiment 1 and in line with the prediction that participants have to safeguard against errors. After that, there is an increase in response hazards that is steeper for consistent than for inconsistent primes, leading to an advantage in mean and median RT. Again, around 400 ms after target onset, this priming effect is gone. As in Experiment 1, early responses are mostly correct when the single prime is consistent, but incorrect when it is inconsistent, showing that early responses are still determined by the prime, not the target.^8^

Let us now look at the double-prime conditions where the P1–P2 SOA is short and the P2–T SOA is long (80/187, second column in Fig. 9), so that the impact of the second prime should be high relative to the first prime. Again, although the very earliest responses occur around the same time in all priming conditions, initial response hazards in CI and IC conditions are lower than in CC and II conditions, reflecting early response competition between both prime-triggered responses (see also the survivor functions). After about 250 ms, both groups begin to differentiate and now follow the order observed in mean RTs: CC, IC, and then CI and II. Again, the most diagnostic information is in the conditional accuracy functions, which show a markedly different pattern than in Experiment 1. The earliest responses are still predominantly correct when both primes are consistent and predominantly incorrect when both primes are inconsistent, showing that the earliest responses are not determined by the target, but by information in the primes. However, conditional accuracy functions for CC and IC are virtually identical, as are those of II and CI. In other words, the earliest systematic responses reflect only the second prime and not the first, probably because the P1–T SOA is too long. However, if observers would respond faster, we believe that the very first responses would reflect the first prime, just as in Experiment 1.

Although these early effects seem to be driven largely by the second prime, comparison with the 133/133 and 187/80 SOA conditions shows that the effects on hazard cannot be attributed to the second prime alone. While the CC, CI, and II condition show highly similar time courses in every condition, this is not true for the IC condition: The longer the first SOA and the shorter the second one, the more delay appears in condition IC compared with CC (the same effect that is evident in average RT). This effect shows that the first prime has an influence on the timing of the response. Moreover, it appears that with shorter P2–T SOA, effects of the second prime become visible in the conditional accuracy functions. Earliest responses are increasingly more incorrect in the IC condition and increasingly more accurate in the CI condition.

#### Event history analysis: Inferential statistics

**Fig. 10:**
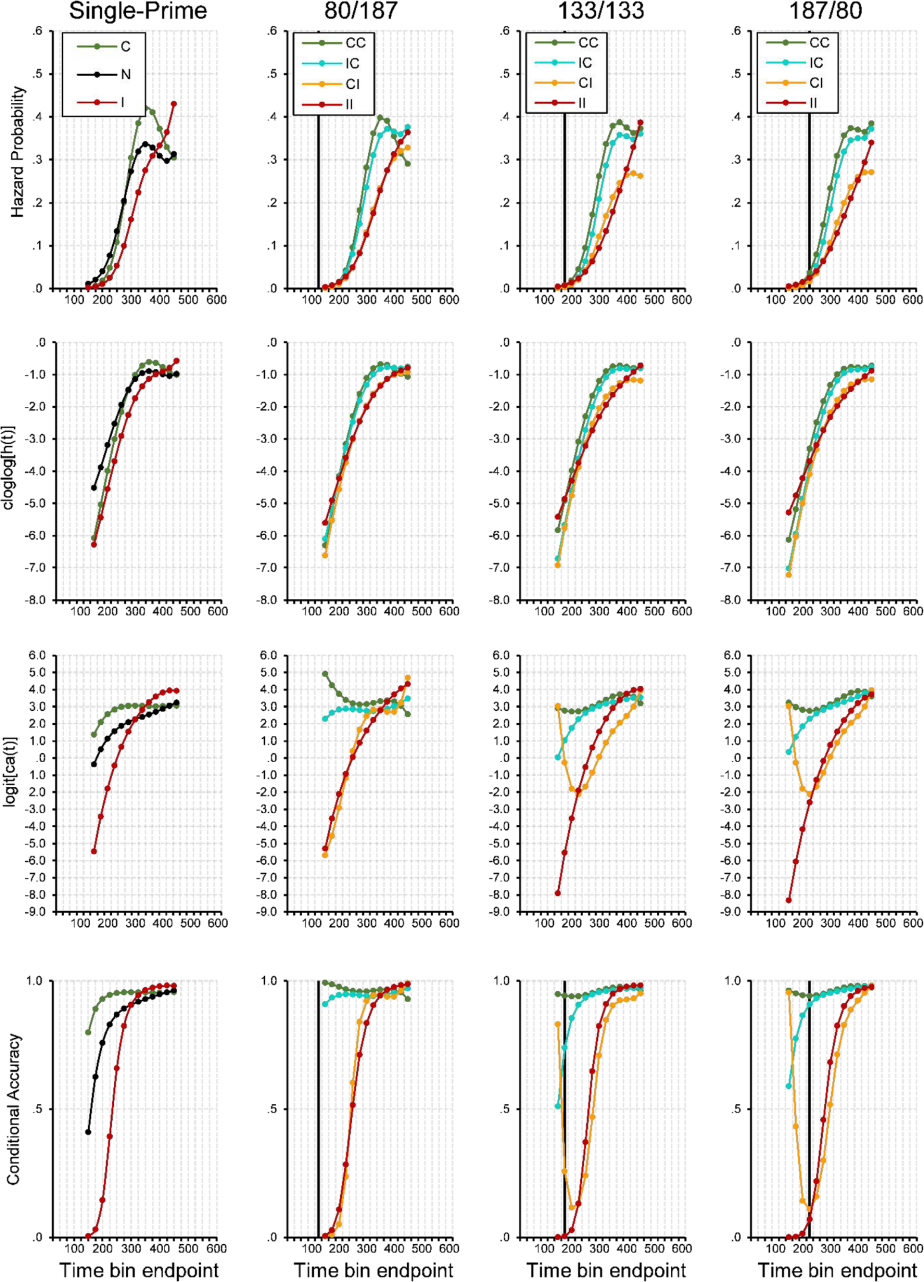
Model predictions. Predicted hazard (first row), cloglog[*h*(t)] (second row), logit[*ca*(t)] (third row), and conditional accuracy functions (fourth row) for trial 1,000 of Experiment 2. Black vertical lines highlight bins at ~275–300 ms after onset of P2

Table 3 shows the selected hazard model, and Table 4 the selected *ca*(t) model. Figure 10 shows predicted (i.e., model-based hazard) cloglog[*h*(t)], logit[*ca*(t)] and conditional accuracy functions for Trial 1,000. The first five parameters in Table 3 model the shape of the cloglog[*h*(t)] function in the baseline condition, IC-187/80 in Trial 1,000 (see Fig. 10, row 2, column 4, blue line). Most importantly, compared with condition IC, changing to CC increases the estimated cloglog-hazard in Bin 275 by .335 units (Parameter 21), changing to CI decreases it by .529 units (Parameter 24), and changing to II decreases it by .569 units (Parameter 16; all *p*s < .0001). While the main effect of CC in Bin 275 decreases over time, the main effects of II and CI increase over time initially (Parameters 17–20, 25–28). Similar to Bin 200 in Experiment 1, in Bin 175 the estimated cloglog-hazard is higher for CC and II than the mixed conditions IC and CI.

The effect of changing the SOA combination from 187/80 to 133/133 is to increase the estimated cloglog-hazard in the early bins (Parameters 32–33). The estimated cloglog-hazard in these bins increases even further when SOA combination is changed to 80/187 (Parameters 29–31). In other words, response occurrence speeds up with increasing P2–T SOA. The remaining interactions between dual-prime (II, CC, CI), SOA, and TIME (Parameters 34–43) mainly reflect a lower cloglog-hazard in Bin 175, especially for SOA combination 80/187.

The first five parameters in Table 4 model the shape of the logit-*ca*(t) function in the baseline condition, IC-187/80 in Trial 1,000 (see Fig. 10, row 3, column 4, blue line). Most importantly, compared with condition IC, changing to CC increases the estimated logit-*ca*(t) in Bin 275 by .111 units (Parameter 15; *p* = .4645), changing to CI decreases it by 3.678 units (Parameter 19; *p* < .0001), and changing to II decreases it by 2.997 units (Parameter 12; *p* < .0001). These main effects of CC, CI, and II in Bin 275 decrease in magnitude over time (Parameters 12–23).

There is no significant main effect of decreasing the P1–P2 SOA from 187 to 133 (Parameters 28–30), but decreasing it to 80 ms increases the estimated logit-*ca*(t) in Bin 200 and decreases the estimated logit-*ca*(t) for bins >300 ms (Parameters 24–27). Finally, there are time-invariant interactions between II and SOA combinations (Parameters 31–32), and time-varying interactions between CI and SOA combinations (Parameters 33–39), which all increase the estimated logit-*ca*(t) in at least some of the bins.

#### Summary

Overall, even with a long P1–T SOA, single-prime conditions produced the common finding in response priming experiments: faster and more accurate responses in consistent trials and slower and less accurate responses in inconsistent trials. Again, when two primes were presented, responses were fastest and most accurate for two consistent primes, and slowest and least accurate for two inconsistent primes. However, under the SOA conditions of Experiment 2, when primes were mixed, we found a clear dominance of the second prime. In particular, CI was almost identical to II, in both response times and error rates. Similarly, IC was almost identical to CC in error rate, but slightly slower. In other words, in both RT and ER the second prime seemed to dominate the response, yet an inconsistent first prime could still slow down response times. This might reflect early response competition due to conflicting prime information in mixed prime conditions.

Again, in order to investigate the temporal dynamics of sequential motor activation, we performed an event history analysis. Altogether, the findings suggest that with prolonged SOAs: (1) The earliest systematic responses were predominantly controlled by the second prime, (2) the slowest responses were controlled by the target, (3) overt responses to the first prime were extremely rare; however, (4) the first prime was able to slow down initial response hazards in mixed prime conditions compared with conditions with identical primes.

## General discussion

The goal of the current study was to investigate (a) whether sequential primes initiate immediate sequential response activation or integrate in a buffer before a response is emitted, (b) whether sequential response activation at short SOAs conforms to the rapid-chase criteria, and (c) how the influence of the first prime changes when the SOAs are prolonged so that participants have to safeguard against early errors from inconsistent primes.

Event history analysis provides substantial evidence that sequential primes initiate strictly sequential response activation at short SOAs (Experiment 1). First, we found that earliest responses were exclusively controlled by the first prime irrespective of the identity or onset time of the second prime, that intermediate responses were influenced by the second prime (with the magnitude and timing of this effect depending on the second prime’s onset time), and that only late responses were controlled by the actual target. This strongly supports the notion of feedforward and sequential activation, and is in line with previous findings that first responses are exclusively triggered by prime properties, independent of the target, and only later responses are influenced by target properties (Eimer & Schlaghecken, 1998; Grainger et al., 2013; Schmidt & Schmidt, 2010; Schmidt, 2002; Schmidt & Schmidt, 2009; Vath & Schmidt, 2007). Thus, the data adhere to the chase criteria proposed by T. Schmidt (2014): (1) The first prime rather than the target or subsequent prime signals determine the onset and initial direction of the response; (2) target and second prime influence the response before it is completed; (3) movement kinematics initially depend on characteristics of the first prime only and are independent of all characteristics of target and subsequent prime signals.

Second, as mentioned before (see Multiple-Prime Paradigm section), a simple feedforward-sweep model seems to account very well for response priming effects at short SOAs (up to 100 ms). However, priming effects at longer SOAs are more plausibly carried by the content of a response buffer that carries information from both primes but is dominated by the second one (Grainger et al., 2013). This notion is supported by our findings. When SOAs were long (Experiment 2), we found that early systematic responses were predominantly triggered by the second prime’s identity and that later responses were triggered by the target’s identity. In contrast to the first experiment, we found that overt responses to the first prime were extremely rare, but we identified an indirect, covert influence of the first prime’s identity on motor response activation, as there were signs of response competition due to conflicting prime information in mixed prime conditions. This strongly suggests that information of a first prime was indeed maintained in a memory buffer and could influence the response that is otherwise dominated by the second prime. Future computational models of decision-making (cf. Mattler & Palmer, 2012; Schmidt & Schmidt, 2018; Schubert, Palazova, & Hutt, 2013; Ulrich, Schröter, Leuthold, & Birngruber, 2015; Vorberg et al., 2003) should test whether the observed hazard and conditional accuracy functions can be simulated with or without a memory buffer.

It is important to point to the different insights that can be gained from an ANOVA on mean correct RT, versus an event history analysis. First, in accordance with the conclusions from previous findings (Breitmeyer & Hanif, 2008; Grainger et al., 2013), a second prime dominates the priming effect in mean correct RTs and ER, at least for short interprime intervals. However, the event history analysis showed that the first prime dominated the motor response in the earliest bins (Experiment 1). Thus, in contrast to Breitmeyer and Hanif (2008), this suggests that the second prime does not update and override the effects of the first prime, but that both prime-triggered motor responses are competing in mixed conditions, and under the right SOA setup, even the first prime is able to dominate the motor response.

Second, when SOAs were long (Experiment 2), we found an even clearer dominance of the second prime since participants seemed to safeguard against early errors provoked by the first prime by waiting out the target. Although response accuracy was entirely dominated by the second prime, RT analysis revealed that an inconsistent first prime in IC conditions could still slow down responses compared with consistent-only conditions. The event history analysis confirmed that early hazards were lower in mixed prime conditions compared with identical prime conditions. We propose that this is due to response competition created by conflicting prime information. Further, this effect increased with prolonged SOAs between primes, again reflecting a reduced dominance of the second prime due to an increase of the first prime’s effect. Thus, the first prime can still influence the motor response with long SOAs. Note that these systematic differences between SOA ranges imply that long and short SOAs should not be mixed within the same experiment, since the presence of long SOAs would enforce a strategy of waiting out the target even in trials where the SOA is short (Schmidt, Haberkamp, & Schmidt, 2011).

When we compare SOA combination 187/80 of Experiment 2 with SOA combination 27/80 of Experiment 1, we see that P2 dominated behavior more in the former than in the latter condition. Therefore, in line with Grainger et al. (2013), we propose that the first prime can influence the response only out of a memory buffer in Experiment 2, since prime information seemed to be kept active for a prolonged period of time without activating a response on its own.

Importantly, we designed our lollipop stimulus in such a way as to minimize masking effects (no spatial overlap) and Simon/flanker effects (prime information is presented at both sides of the target). However, it is unclear if active response inhibition was playing a role in the generation of the behavior in Experiment 2. Panis and Schmidt (2016) and Schmidt, Hauch, and Schmidt (2015) showed that a second stimulus can trigger active and selective inhibition of the response triggered by a first stimulus, within about 360 ms. For example, for SOA combinations 133/133 and 187/80 we see that CI has a lower conditional accuracy than II for bins after 225 ms. This might be caused by active inhibition of the first compatible response, creating an even stronger activation of the incompatible response channel in condition CI than in II. Future modeling studies should investigate this issue further.

More generally, the information obtained from an event history analysis can provide strong constraints for computational models of the underlying sensory integration, decision, and cognitive control processes (Panis, Moran, Wolkersdorfer, & Schmidt, 2020). For example, existing models differ in (a) whether sensory integration is perfect (e.g., the drift-diffusion model; Ratcliff & Rouder, 1998) or leaky (e.g., the leaky competing accumulator model of Usher & McClelland, 2001), (b) whether the response criterion is fixed (e.g., Poisson accumulator models; Schmidt & Schmidt, 2018; Schubert et al., 2013; Vorberg et al., 2003) or variable (the urgency gating model of Cisek, Puskas, & El-Murr, 2009), and (c) whether classic computational principles (e.g., the Bayesian reader model of Norris, 2006) or dynamic principles (e.g., the dynamic field theory of Schöner, Spencer,, & The DFT Research Group, 2016) are used (e.g., see Carland, Thura, & Cisek, 2019, for a discussion of these issues). Comparing empirical and simulated data from such models using event history analysis will allow future studies to better select between and validate the different computational models available in the literature.

While behavioral experiments are informative, they allow only indirect inferences about the underlying neural correlates. Ultimately, one wants to complement the behavioral data with physiological data such as EEG, fMRI, single-cell data, and so forth. Note that hazard modeling allows incorporating time-varying explanatory covariates such as heart rate, EEG signal amplitude, and gaze location (Allison, 2010), which is useful for cognitive psychophysiology (Meyer, Osman, Irwin, & Yantis, 1988).

In summary, the current study provides substantial evidence that sequential primes actually initiate sequential response activation, and that this sequence conforms to the chase criteria at short SOAs. However, when SOAs are prolonged participants have to delay their responses, the first prime seems to influence responses out of a memory buffer.

### Open practices statement

All data, materials and analyses are available from the authors upon request. All independent and dependent variables are identified. Preregistration was not employed.
